# Associated Factors and Dose–Response of Steroid‐Induced Diabetes Mellitus in Dermatology Inpatients: A Retrospective Study

**DOI:** 10.1155/drp/4271696

**Published:** 2026-08-19

**Authors:** Jingxi Zhang, Huimin Tang, Shantao Qiu, Yao Sun, Guan Jiang

**Affiliations:** ^1^ Department of Dermatology, The Affiliated Hospital of Xuzhou Medical University, 99 West Huai Hai Road, Xuzhou 221002, Jiangsu, China, xzmc.edu.cn; ^2^ Xuzhou Medical University, 209 Tong Shan Road, Xuzhou 221004, Jiangsu, China, xzmc.edu.cn

**Keywords:** associated factors, dermatological disease, dose–response relationship, glucocorticoids, steroid-induced diabetes mellitus

## Abstract

**Objective:**

To investigate the prevalence and associated factors of steroid‐induced diabetes mellitus (SDM) in dermatology inpatients receiving systemic glucocorticoid (GC) therapy, with particular focus on characterizing the dose–response relationship.

**Study Design:**

Retrospective observational cohort study.

**Place and Duration of Study:**

Department of Dermatology, The Affiliated Hospital of Xuzhou Medical University, Xuzhou, China, from August 2019 to October 2024.

**Methodology:**

A total of 293 patients treated with GCs were included, with 42 classified as having SDM and 251 as controls based on blood glucose monitoring. Data such as demographics, GC dosage, laboratory indices, and follow‐up outcomes were analyzed by SPSS 26.0, R 4.4.1, and logistic regression analysis was applied to explore factors associated with the development of SDM. We modeled the nonlinear dose–response between average daily GC dose (methylprednisolone‐equivalent) and SDM using multivariable logistic regression with restricted cubic splines, setting 0.6 mg/kg/day as the reference. All tests were two‐sided, *p* < 0.05 indicates that the difference is statistically significant.

**Results:**

Among 293 patients, 42 (14.3%) developed SDM and 17 (5.8%) had impaired glucose regulation. SDM cases were older and had higher BMI, hypertension, family history of diabetes, higher average daily GC dose, and more frequent immunosuppressant use. Multivariate regression identified family history of diabetes (OR: 11.16), immunosuppressant use (OR: 3.48), average daily GC dose (OR: 29.13), and serum uric acid (OR: 1.007) as independent factors associated with SDM. The dose–response between average daily GC dose and SDM was significant (Wald *χ* ^2^ = 18.48, df = 3, *p* = 0.0004); risk rose steeply beyond 1.0 mg/kg/day (e.g., 1.2 mg/kg/day: OR: 3.94, 95% CI: 1.44–10.66).

**Conclusion:**

SDM is prevalent among dermatology inpatients receiving GCs. High‐risk patients may benefit from closer glucose monitoring and individualized dose adjustment; prospective studies are warranted to confirm these associations.

## 1. Introduction

Glucocorticoids (GCs) are extensively employed in clinical practice due to their potent anti‐inflammatory and immunosuppressive properties. However, prolonged or high‐dose GC therapy can lead to steroid‐induced diabetes mellitus (SDM), a condition resembling Type 2 diabetes that involves pancreatic β‐cell dysfunction and insulin resistance [1, 2]. A meta‐analysis indicated that the rates of GC‐induced hyperglycemia and diabetes were 32.3% and 18.6%, respectively [3, 4]. Moreover, SDM has been linked to adverse outcomes, including extended hospital stays, increased susceptibility to infections, and elevated mortality rates [5, 6].

Given the pathophysiological similarities between SDM and Type 2 diabetes, biomarkers such as red blood cell distribution width (RDW), serum uric acid (SUA), and cystatin C (Cys C) have been proposed as potential factors associated with SDM [7, 8]. In dermatology, systemic GCs are commonly administered to manage autoimmune and allergic skin conditions, including pemphigus and lupus erythematosus. However, limited research has focused on the incidence and associated factors of SDM in this specific patient population.

While higher GC exposure is linked to hyperglycemia, the shape of the dose–risk relationship may be nonlinear in clinical cohorts. Restricted cubic splines (RCSs) enable quantification of potential thresholds and clinically interpretable risk contrasts at dosing values commonly used in dermatology wards. We, therefore, examined not only independent factors associated with SDM but also the GC dose–response curve using RCS to identify inflection points relevant to inpatient management.

Accordingly, we conducted a retrospective cohort study in a dermatology inpatient setting to clarify the burden and determinants of SDM under real‐world GC use. Specifically, we aimed to identify independent clinical factors associated with SDM using multivariable logistic regression. We also modeled the dose–response relationship between average daily GC dose (methylprednisolone‐equivalent) and SDM using RCSs, anchoring risk estimates at 0.6 mg/kg/day (near the cohort median). By leveraging routinely collected ward‐level data, our goal was to provide actionable risk indicators and dose thresholds to guide glucose‐monitoring intensity and inform dose titration in dermatology practice.

Our research group previously reported a machine‐learning prediction model for SDM in the same cohort [9]; the present study instead focuses on etiological factors and the dose–response relationship using conventional statistical methods.

## 2. Methodology

Patients were enrolled by consecutive sampling, including all eligible dermatology inpatients who met the inclusion and exclusion criteria during the observation window (August 2019–October 2024), to minimize selection bias. Data extraction and statistical analysis were conducted between November 2024 and March 2025.

A total of 293 dermatology inpatients treated with GCs between August 2019 and October 2024 were included. The inclusion criteria were (1) age > 12 years; (2) diagnosed with severe erythema multiforme, drug eruption, pemphigus, bullous pemphigoid, lupus erythematosus, or dermatomyositis and treated with standardized GC therapy; (3) intravenous GC treatment with at least 30 mg/day of methylprednisolone for at least 2 days; (4) no prior history of diabetes or abnormal blood glucose before GC treatment; however, HbA1c and oral glucose tolerance tests were not routinely performed at baseline and, therefore, undiagnosed prediabetes or latent diabetes could not be completely excluded; and (5) follow‐up data for more than 3 months, including fasting and postprandial blood glucose (PBG). Exclusion criteria included (1) prior glucose metabolism disorders or GC use; (2) use of medications that could raise blood glucose, such as antipsychotics or calcineurin inhibitors; (3) body mass index (BMI) ≥ 28 kg/m^2^ or metabolic syndrome; and (4) severe liver or pancreatic diseases affecting blood glucose. SDM was diagnosed based on fasting blood glucose (FBG) ≥ 7.0 mmol/L, PBG ≥ 11.1 mmol/L, or random blood glucose ≥ 11.1 mmol/L after GC treatment. Steroid‐induced hyperglycemia was defined as elevated blood glucose levels that did not meet the diagnostic criteria for diabetes (FBG 6.1–6.9 mmol/L or PBG 7.8–11.0 mmol/L), particularly when transient. Impaired glucose regulation (IGR) was defined as intermediate hyperglycemia, including impaired fasting glucose and/or impaired glucose tolerance within the above ranges. It should be noted that SDM was defined according to blood glucose criteria (peak glucose values) rather than treatment modality. Some patients meeting the diagnostic criteria for SDM subsequently achieved normoglycemia with lifestyle modification alone, highlighting the potential overlap between transient steroid‐induced hyperglycemia and SDM during the acute phase.

Blood glucose monitoring was performed as part of routine inpatient management. FBG was generally assessed every 1–3 days during hospitalization, with additional postprandial or random glucose measurements conducted when clinically indicated. In patients with elevated glucose levels, repeat measurements were performed to confirm the abnormal findings and reduce the likelihood of transient fluctuations or misclassification.

Due to the retrospective design, a strictly predefined monitoring protocol was not available; however, glucose surveillance was routinely implemented in a broadly consistent manner across patients receiving systemic GC therapy.

No predefined cutoff time was set for the development of SDM. SDM was identified based on glucose criteria occurring at any time after initiation of GC therapy during hospitalization and follow‐up. All patients were followed for at least 3 months, allowing adequate detection of both early‐onset and relatively delayed cases.

Data were collected on demographics (age, sex, height, weight, hypertension, family history of diabetes, smoking, and alcohol use), GC use (type, dose, and duration), and immunosuppressant use.

All baseline demographic variables (age, BMI, hypertension, and family history of diabetes) were ascertained at admission, and laboratory indices were obtained at or before the initiation of GC therapy. Thus, all candidate variables temporally preceded the occurrence of the SDM outcome.

Laboratory data included RDW, liver function (ALT, AST, and GGT), renal function (BUN, SUA, SCr, and Cys C), FBG, lipids (TC, TG, HDL‐C, and LDL‐C), and serum magnesium. The average daily GC dose (methylprednisolone‐equivalent, mg/kg) was calculated as the cumulative dose prior to SDM diagnosis divided by the corresponding number of exposure days; immunosuppressant use likewise refers to exposure preceding SDM diagnosis, ensuring that exposures temporally preceded the outcome and avoiding reverse causation. Data collection was performed by the investigators. Missing data were present in 37 patients (12.6%). Variables with missing values included GGT (6.1%), BUN (4.1%), SCr (3.8%), SUA (6.8%), Cys C (8.2%), TC (5.1%), TG (4.8%), HDL‐C (5.1%), and BMI (3.1%). Missing values for continuous variables were imputed using mean imputation. For variables with less than 5% missing data, nearest‐neighbor imputation was additionally performed as a sensitivity analysis, which yielded consistent results and supported the robustness of the imputation strategy.

Statistical analyses were performed using SPSS 26.0 and R 4.4.1. Normality of continuous variables was assessed with the Shapiro–Wilk test, and all variables were nonnormally distributed (*p* < 0.05). Continuous data are therefore expressed as median (IQR) and were compared between groups using the Mann–Whitney *U* test. Categorical variables were analyzed using Fisher’s exact test. Variables with an unadjusted *p* < 0.20 in univariate logistic regression were entered into the multivariable logistic regression model to identify factors independently associated with SDM. All statistical tests were two‐tailed, and a *p* < 0.05 was considered statistically significant.

To characterize potential nonlinearity between average daily GC dose and SDM, we fitted a multivariable logistic regression model with RCSs (four knots placed at dose quantiles). The reference dose was 0.6 mg/kg/day (OR = 1), which was close to the cohort median. Covariates included age, BMI, family history of diabetes, immunosuppressant use, and hypertension; SUA was modeled with a 3‐knot spline to account for metabolic/renal confounding. Predictions were generated for a “typical patient” (continuous covariates at medians; binary covariates set to “No”), with 95% CIs. We plotted ORs on a log10‐scaled *x*‐axis and reported Wald *χ* ^2^ tests for the overall and nonlinear components.

## 3. Results

**FIGURE 1:**
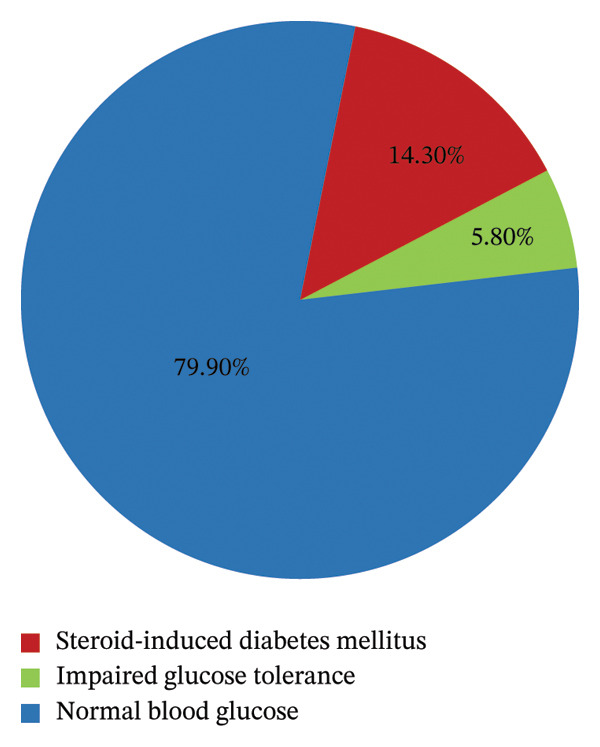
Blood glucose status of 293 patients after GC treatment.

A total of 293 dermatology inpatients were included, comprising 81 cases of severe erythema multiforme, 52 lupus erythematosus, 27 bullous pemphigoid, 41 dermatomyositis, 19 pemphigus, and 73 severe drug eruptions. Among them, 59 developed steroid‐induced hyperglycemia, with 42 cases of SDM (14.3%) and 17 with IGR (5.8%) (Figure 1).

More than half (54.8%, 23/42) of SDM cases occurred within the first month after initiation of GC therapy. Among the 42 SDM cases, 25 were asymptomatic and were identified through routine glucose monitoring during hospitalization, while 17 experienced polydipsia and polyuria. One patient developed diabetic ketoacidosis. Among the 42 patients with SDM, 12 achieved normalization of blood glucose through diet and exercise alone, suggesting that these cases may represent transient steroid‐induced hyperglycemia within the overall SDM cohort. The remaining 30 patients required oral hypoglycemic agents or insulin therapy, with good prognosis and no complications or deaths reported during follow‐up.

As shown in Table 1, significant differences were found between the SDM group and the control group in age, BMI, history of hypertension, family history of diabetes, and immunosuppressant use (*p* < 0.05). As shown in Table 2, laboratory results showed that SUA levels were significantly higher in the SDM group compared to the control group. In addition, levels of GGT, BUN, SCr, and Cys C were also elevated in the SDM group, while HDL levels were lower (*p* < 0.05).

**TABLE 1:** Comparison of the demographic data of the two groups.

Observation metrics	SDM group (*n* = 42)	Control group (*n* = 251)	*p* value
Age (years), median [IQR]	54.00 [45.50, 62.00]	47.00 [33.00, 62.00]	0.017
BMI (kg/m^2^), median [IQR]	24.87 [23.04, 26.78]	23.53 [21.16, 25.71]	0.008
Male: Female	16:26	97:154	1.000
Hypertensive disease, *n* (%)	13 (31.0)	37 (14.7)	0.015
Family history of diabetes, *n* (%)	16 (38.1)	10 (4.0)	< 0.001
Smoking history, *n* (%)	5 (11.9)	18 (7.2)	0.347
Alcohol consumption history, *n* (%)	4 (9.5)	13 (5.2)	0.281
Immunosuppressant use, *n* (%)	24 (57.1)	39 (15.5)	< 0.001

*Note:* *p* value: continuous variables were compared with the Mann–Whitney *U* test; categorical variables with Fisher’s exact test. SDM, steroid‐induced diabetes mellitus; IQR, interquartile range.

Abbreviation: BMI, body mass index.

**TABLE 2:** Comparison of laboratory examination indexes between the two groups.

Observation metrics	SDM group (*n* = 42)	Control group (*n* = 251)	*p* value
GGT (U/L), median [IQR]	31.50 [21.25, 50.50]	23.00 [14.00, 39.00]	0.004
BUN (mmol/L), median [IQR]	6.78 [4.67, 8.73]	4.59 [3.79, 6.00]	< 0.001
SCr (μmol/L), median [IQR]	63.00 [49.75, 83.50]	54.00 [44.00, 63.50]	0.002
SUA (μmol/L), median [IQR]	293.00 [239.50, 370.75]	235.00 [182.00, 305.00]	< 0.001
Cys C (mg/L), median [IQR]	1.17 [0.87, 1.40]	0.93 [0.78, 1.09]	< 0.001
FBG (mmol/L), median [IQR]	9.32 [8.01, 10.43]	4.78 [4.34, 5.36]	< 0.001
TC (mmol/L), median [IQR]	5.15 [4.05, 5.74]	4.12 [3.46, 4.94]	< 0.001
TG (mmol/L), median [IQR]	1.66 [1.14, 2.49]	1.2 [0.88, 1.64]	< 0.001
HDL‐C (mmol/L), median [IQR]	1.07 [0.83, 1.47]	1.31 [1.06, 1.65]	0.005

*Note:* *p* value: continuous variables were compared with the Mann–Whitney *U* test. SDM, steroid‐induced diabetes mellitus; IQR, interquartile range; SCr, serum creatinine; Cys C, cystatin C; TG, triglycerides.

Abbreviations: BUN, blood urea nitrogen; FBG, fasting blood glucose; GGT, gamma‐glutamyl transferase; HDL‐C, high‐density lipoprotein cholesterol; SUA, serum uric acid; TC, total cholesterol.

In Table 3, the SDM group had a significantly higher average daily GC dose (0.89 [0.64, 1.29] mg/kg) compared to the control group (0.64 [0.50, 0.84] mg/kg, *p* < 0.001), though starting dose, maximum daily dose, and treatment duration showed no significant differences.

**TABLE 3:** Comparison of GC usage between the two groups.

Observation metrics	SDM group (*n* = 42)	Control group (*n* = 251)	*p* value
Average daily dose (mg/kg), median [IQR]	0.89 [0.64, 1.29]	0.64 [0.50, 0.84]	**< 0.001**
The starting dose (mg/day), median [IQR]	54.00 [40.00, 60.00]	40.00 [32.00, 60.00]	0.299
Maximum daily dose (mg/kg), median [IQR]	0.86 [0.68, 1.07]	0.74 [0.57, 1.00]	0.072
Duration (d), median [IQR]	6.50 [5.00, 9.75]	8.00 [6.00, 11.00]	0.254

*Note:* *p* value: Continuous variables were compared with the Mann–Whitney *U* test. SDM, steroid‐induced diabetes mellitus; IQR, interquartile range; GC, glucocorticoids. Bold value represents *p* < 0.001, indicating statistically significant intergroup differences.

As shown in Table 4, *p* values were calculated using univariate logistic regression. Variables with an unadjusted *p* < 0.20 were subsequently included in the multivariate analysis. In Table 5, *p* values were derived from multivariate logistic regression, including variables with unadjusted *p* < 0.20 in the univariate analysis. In this multivariate model, family history of diabetes (*p* < 0.001), immunosuppressant usage (*p* = 0.014), average daily GC dose (*p* < 0.001), and SUA level (*p* = 0.029) were independently associated with SDM.

**TABLE 4:** Univariate logistic regression analysis of associated factors for steroid‐induced diabetes mellitus.

Variable	OR	95% confidence interval		*p* value
		Lower limit	Upper limit	
Age (years)	1.023	1.004	1.043	0.021
BMI (kg/m^2^)	1.123	1.027	1.231	0.011
Family history of diabetes	14.831	6.205	37.183	< 0.001
Hypertensive disease	2.593	1.206	5.370	0.012
Use of immunosuppressant	7.248	3.623	14.792	< 0.001
Average daily GC dose (mg/kg)	27.220	8.107	101.230	< 0.001
TC (mmol/L)	1.442	1.132	1.857	0.003
TG (mmol/L)	1.835	1.350	2.555	< 0.001
HDL‐C (mmol/L)	0.373	0.155	0.844	0.022
SCr (μmol/L)	1.007	1.000	1.015	0.040
Cys C (mg/L)	2.825	1.546	5.645	0.002
BUN (mmol/L)	1.201	1.096	1.325	< 0.001
SUA (μmol/L)	1.008	1.004	1.011	< 0.001
GGT (U/L)	1.003	0.998	1.008	0.154

*Note:* Data are odds ratios (OR) with 95% confidence intervals. *p* values were obtained from two‐sided Wald *χ* ^2^ tests in univariate logistic regression. GC, glucocorticoids; TG, triglycerides; SCr, serum creatinine; CysC, cystatin C.

Abbreviations: BMI, body mass index; BUN, blood urea nitrogen; GGT, gamma‐glutamyl transferase; HDL‐C, high‐density lipoprotein cholesterol; OR, odds ratio; SUA, serum uric acid; TC, total cholesterol.

**TABLE 5:** Multivariate logistic regression analysis of associated factors for steroid‐induced diabetes mellitus.

Variable	OR	95% confidence interval		*p* value
		Lower limit	Upper limit	
Age (years)	1.022	0.991	1.056	0.167
BMI (kg/m^2^)	1.075	0.941	1.232	0.287
Family history of diabetes	11.160	2.931	44.913	**< 0.001**
Hypertensive disease	0.570	0.138	2.009	0.405
Use of immunosuppressant	3.483	1.268	9.535	**0.014**
Average daily GC dose (mg/kg)	29.125	5.670	179.782	**< 0.001**
GGT (U/L)	1.000	0.984	1.008	0.643
BUN (mmol/L)	1.058	0.871	1.269	0.563
SCr (μmol/L)	0.993	0.974	1.006	0.373
SUA (μmol/L)	1.007	1.001	1.013	**0.029**
Cys C (mg/L)	1.673	0.562	3.645	0.218
TC (mmol/L)	1.202	0.857	1.685	0.282
TG (mmol/L)	1.414	0.770	2.417	0.234
HDL‐C (mmol/L)	0.428	0.136	1.178	0.120

*Note:* Data are adjusted odds ratios with 95% confidence intervals from the multivariable logistic model. *p* values were obtained from two‐sided Wald *χ* ^2^ tests on the partial regression coefficients. GC, glucocorticoids; SCr, serum creatinine; TG, triglycerides. Bold value represents *p* < 0.05, indicating statistically significant intergroup differences.

Abbreviations: BMI, body mass index; BUN, blood urea nitrogen; GGT, gamma‐glutamyl transferase; HDL‐C, high‐density lipoprotein cholesterol; OR, odds ratio; SUA, serum uric acid; TC, total cholesterol.

**FIGURE 2:**
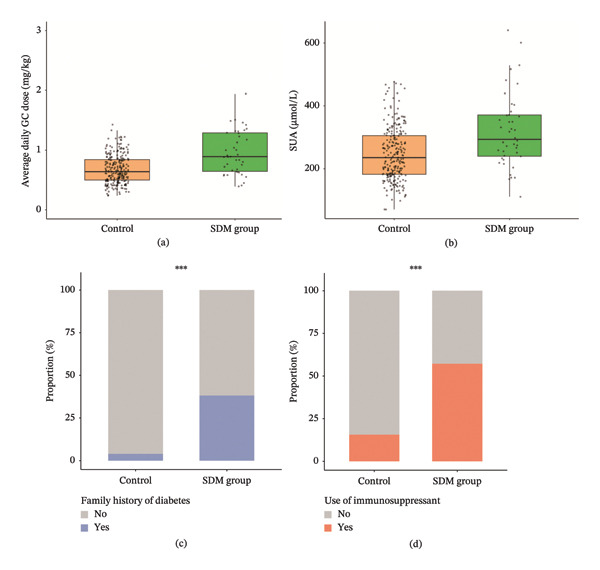
Comparison of selected associated factors between SDM and non‐SDM patients.

Logistic regression identified family history of diabetes, immunosuppressant usage, average daily GC dose, and SUA as independent factors associated with SDM (*p* < 0.05). Figures illustrating the differences in GC dose, SUA levels, and the proportions of family history and immunosuppressant use between the two groups are shown in Figure 2.

**FIGURE 3:**
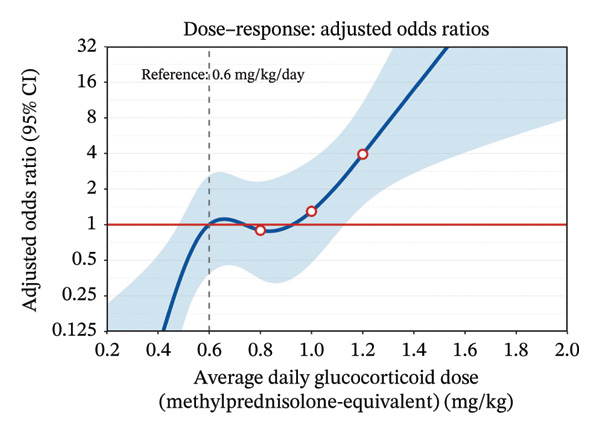
Dose–response curve of average daily GC dose and the risk of SDM based on the multivariable logistic regression model with RCS.

In the multivariable RCS model, the overall association between GC dose and SDM was significant (Wald *χ* ^2^ = 18.48, df = 3, *p* = 0.0004). Using 0.6 mg/kg/day as the reference, adjusted ORs for a typical patient showed a modest dip near 0.8 mg/kg/day and then a steeper increase once the dose exceeded approximately 1.0 mg/kg/day: 0.4 mg/kg/day, OR: 0.09 (95% CI: 0.01–0.55); 0.8 mg/kg/day, OR: 0.90 (0.35–2.31); 1.0 mg/kg/day, OR: 1.30 (0.48–3.55); 1.2 mg/kg/day, OR: 3.94 (1.44–10.66) (Figure 3).

## 4. Discussion

GCs are widely prescribed in dermatology due to their potent anti‐inflammatory and immunosuppressive properties. However, their metabolic side effects, particularly SDM, pose significant clinical concerns. In this study, SDM occurred in 14.3% of the hospitalized dermatology patients receiving systemic GCs, a rate consistent with previously reported ranges [10, 11]. SDM often develops rapidly after starting GC treatment [4, 12], with more than half (54.8%) of the cases occurring within the first month after initiation of GC therapy, consistent with the findings of Gonzalez et al., who reported that diabetes most frequently developed during the second to fourth week [13]. Given the variability in onset timing across studies, we did not impose a strict temporal cutoff, in order to avoid underestimation of SDM cases. This finding highlights the need for heightened awareness and early detection of SDM in dermatological practice.

In previous studies, several factors associated with SDM have been identified earlier, including age and BMI, with older age (≥ 60 years) and higher BMI (> 30 kg/m^2^) independently associated with increased risk [4, 14, 15]. Older age is linked to pancreatic β‐cell decline, reduced insulin secretion, and decreased insulin sensitivity in tissues such as muscles, fat, and liver. [14] Additionally, obesity exacerbates insulin resistance through the release of proinflammatory factors like TNF‐α and IL‐6, damaging insulin signaling pathways and pancreatic β‐cells [16].

In our study, multivariate analysis revealed four independent factors associated with SDM: a family history of diabetes, immunosuppressant use, higher average daily GC dose, and elevated SUA. The strong association with family history supports the role of genetic susceptibility in GC‐related glucose dysregulation. Similarly, the concurrent use of immunosuppressants, especially agents such as mycophenolate and cyclophosphamide, may compound the diabetogenic effects of GCs, likely through additive impacts on pancreatic β‐cell function or systemic inflammation.

The observed association between higher average daily GC dose and SDM risk suggests a potential dose‐dependent relationship in GC‐related metabolic effects. This finding aligns with prior pharmacodynamic studies showing that high‐dose GCs promote hepatic gluconeogenesis, impair peripheral glucose uptake, and directly injure pancreatic β‐cells [17].

Beyond identifying independent predictors, our spline analysis clarified the shape of GC‐related risk: SDM probability rose modestly at moderate doses but increased more steeply once the average daily GC dose exceeded approximately 1.0 mg/kg/day. This inflection point is biologically plausible, given dose‐dependent hepatic gluconeogenesis and peripheral insulin resistance under systemic GC exposure, and provides a practical target for dose titration and glucose‐monitoring intensity. The spline‐based ORs are expressed relative to a clinical reference (0.6 mg/kg/day) in a typical patient and complement the per‐unit effect estimates from the multivariable model. These findings suggest an association between higher GC exposure and increased SDM risk beyond approximately 1.0 mg/kg/day, which may help identify patients who warrant closer glycemic monitoring.

Contrary to some earlier reports, we did not observe significant associations between SDM and gender, smoking, or alcohol consumption. These inconsistencies may be due to differences in study populations, sample sizes, or criteria for metabolic comorbidities. Notably, age and BMI although significant in univariate analysis did not retain significance in the multivariate model, suggesting that their effects may be mediated through other variables such as GC dose or baseline metabolic reserve.

This study has several strengths, including its focus on a unique population (dermatology inpatients), detailed clinical and laboratory data, and rigorous statistical adjustments for multiple comparisons. However, several limitations must be acknowledged. First, the retrospective design precludes causal inference and may introduce selection bias. Thyroid function and hypothyroidism status were not systematically evaluated, which may confound glucose metabolism. In addition, disease‐specific subgroup analyses were not performed due to limited sample size within each subgroup, which may limit the identification of disease‐specific risks. Second, the relatively small number of SDM cases (*n* = 42) limits statistical power and generalizability. Third, the lack of comprehensive glucose monitoring (e.g., HbA1c and OGTT) may have led to an underestimation of SDM incidence, particularly among asymptomatic patients. In addition, glucose monitoring was not based on a strictly standardized protocol across all patients due to the retrospective nature of the study. Although blood glucose was routinely assessed in clinical practice, variations in monitoring frequency may have introduced potential surveillance bias, especially among asymptomatic individuals. This should be considered when interpreting the observed associations. Nevertheless, as glucose monitoring is a standard component of inpatient care for patients receiving systemic GCs, the overall impact of such bias may be limited.

Furthermore, baseline glycemic assessment may have been incomplete. Although patients with a known history of diabetes or abnormal baseline glucose were excluded, HbA1c and oral glucose tolerance tests were not routinely performed. Therefore, undiagnosed prediabetes or latent diabetes could not be entirely ruled out, and some cases classified as steroid‐induced diabetes may have represented previously unrecognized dysglycemia.

Additionally, some patients experienced normalization of blood glucose with lifestyle modification alone, suggesting that a subset of cases may represent transient steroid‐induced hyperglycemia. As classification in this study was based on peak glucose values recorded during hospitalization, some overlap between transient hyperglycemia and SDM cannot be fully excluded. Finally, the absence of long‐term follow‐up limits our ability to assess chronic complications or sustained remission.

Future prospective studies should aim to validate these findings in larger, multicenter cohorts and incorporate comprehensive metabolic assessments. In particular, the longitudinal tracking of glycemic outcomes and GC tapering schedules would provide valuable insight into the reversibility and prognosis of SDM. Moreover, integrating machine learning models could facilitate individualized SDM risk prediction, enhancing clinical decision‐making.

## 5. Conclusion

In this retrospective cohort of dermatology inpatients on systemic GCs, SDM was common and independently associated with family history of diabetes, immunosuppressant use, higher average daily GC dose, and elevated SUA. The spline‐modeled dose–response indicated a steeper risk rise beyond 1.0 mg/kg/day (methylprednisolone‐equivalent), supporting intensified glucose monitoring and dose minimization when clinically feasible. Prospective, multicenter validation is warranted.

## Author Contributions

Jingxi Zhang and Huimin Tang: performed statistical analysis of data and manuscript writing and responsible for the accuracy and integrity of the study.

Shantao Qiu and Yao Sun: manuscript writing and responsible for the accuracy and integrity of the study.

Guan Jiang: literature search, did review, and gave final approval of the manuscript.

## Funding

This research received no funding.

## Disclosure

This study shares the same cohort with a companion manuscript addressing a different research question. The two papers have distinct objectives, analyses, and figures, and minimal overlap beyond baseline description.

## Ethics Statement

This study was approved by the Medical Ethics Committee of the Affiliated Hospital of Xuzhou Medical University (approval no. XYFY2022‐KL425‐02) prior to initiation and was conducted in strict accordance with the principles of the Declaration of Helsinki concerning research involving human subjects.

## Consent

All adult participants provided written informed consent. For adolescent participants, written informed consent was obtained from their parents or legal guardians. All participants’ privacy and confidentiality were strictly protected.

## Conflicts of Interest

The authors declare no conflicts of interest.

## Data Availability

The datasets generated or analyzed during the current study are available from the corresponding author on reasonable request.
